# Validation of the in vitro comet assay for DNA cross-links and altered bases detection

**DOI:** 10.1007/s00204-021-03102-3

**Published:** 2021-07-01

**Authors:** Damián Muruzabal, Julen Sanz-Serrano, Sylvie Sauvaigo, Bertrand Treillard, Ann-Karin Olsen, Adela López de Cerain, Ariane Vettorazzi, Amaya Azqueta

**Affiliations:** 1 Department of Pharmacology and Toxicology, University of Navarra, C/Irunlarrea 1, 31009 Pamplona, Spain; 2 LXRepair, Biopolis, 5 Avenue du Grand Sablon, 38700 La Tronche, France; 3 Section of Molecular Toxicology, Department of Environmental Health, Norwegian Institute of Public Health, Skøyen, PO Box 222, 0213 Oslo, Norway; 4 IdiSNA, Navarra Institute for Health Research, Pamplona, Spain

**Keywords:** Comet assay, Cross-links, Oxidized bases, Alkylated bases, Mechanism of action, In vitro

## Abstract

Mechanistic toxicology is gaining weight for human health risk assessment. Different mechanistic assays are available, such as the comet assay, which detects DNA damage at the level of individual cells. However, the conventional alkaline version only detects strand breaks and alkali-labile sites. We have validated two modifications of the in vitro assay to generate mechanistic information: (1) use of DNA-repair enzymes (i.e., formamidopyrimidine DNA glycosylase, endonuclease III, human 8-oxoguanine DNA glycosylase I and human alkyladenine DNA glycosylase) for detection of oxidized and alkylated bases as well as (2) a modification for detecting cross-links. Seven genotoxicants with different mechanisms of action (potassium bromate, methyl methanesulfonate, ethyl methanesulfonate, hydrogen peroxide, cisplatin, mitomycin C, and benzo[a]pyrene diol epoxide), as well as a non-genotoxic compound (dimethyl sulfoxide) and a cytotoxic compound (Triton X-100) were tested on TK-6 cells. We were able to detect with high sensitivity and clearly differentiate oxidizing, alkylating and cross-linking agents. These modifications of the comet assay significantly increase its sensitivity and its specificity towards DNA lesions, providing mechanistic information regarding the type of damage.

## Introduction

During the last years, the Adverse Outcome Pathway (AOP) concept has arisen as a pragmatic tool in the toxicological evaluation of all kind of chemicals based on a more human relevant mechanistic toxicology. AOPs are conceptual constructs aimed to support risk assessment by understanding the mechanism linking a molecular initiating event (MIE, e.g*.,* binding to an enzyme) with an adverse outcome (AO, e.g., heritable mutations or cancer), through a progression of measurable biological changes, known as key events (KE, e.g., DNA alkylation) (Ankley et al. 2010; Leist et al. 2017). KE are considered as relevant factors and potential endpoints in decision-making processes for hazard identification (Leist et al. 2017). Indeed, the Organisation for Economic Co-operation and Development (OECD) supports the AOP concept and has prepared a workplan for its development (OECD 2020).

In this context, the detection and measurement of KE with reliable tools and methods is of great relevance. Regarding genotoxicity assessment, different assays are available for measuring KE, such as the alkaline comet assay (single cell gel electrophoresis) which is a method to measure DNA damage levels, particularly strand breaks (SB) and alkali-labile sites (ALS) (apurinic/apyrimidinic-AP-sites or baseless sugars), at the level of individual cells. Its versatility makes it a widely used technique, as it can be applied to any eukaryotic cell type, including disaggregated tissues from which single cells or nuclear suspensions can be obtained (Vasquez 2012; Azqueta and Collins 2013; Jackson et al. 2013; Asare et al. 2016). Indeed, the technique is used in different scientific fields, such as human and environmental biomonitoring or in vivo and in vitro genotoxicity testing of chemicals and nanoparticles among others.

The comet assay is relatively simple, and it was developed on its alkaline version by Singh and colleagues in 1988 (Singh et al. 1988). In brief, cells are embedded in agarose on a microscope slide and lysed with detergent and high salt concentration to remove cell membranes and soluble components leaving a nucleoid, consisting of supercoiled DNA attached to a matrix. Then, the lysed cells are subjected to alkaline conditions to unwind DNA followed by electrophoresis. If the DNA integrity of a nucleoid is disrupted by a SB, supercoiling is relaxed and part of the DNA will extend due to the electrophoretic force giving a comet-like image when stained with a nucleic acid specific dye and evaluated by fluorescence microscopy; whereas if DNA remains undamaged, supercoiling is preserved and, therefore, no comet tail is formed (Collins 2004).

From the regulatory point of view, the in vivo version of the comet assay has been validated, and the OECD published an in vivo Mammalian Alkaline Comet Assay Guideline (TG 489) (OECD 2016). The in vitro version of the comet assay does not have an OECD guideline; however, its combination with a 3D skin model was validated in a study led by Cosmetics Europe with the support of the European Union Reference Laboratory for Alternatives to Animal Testing (EURL-ECVAM) and is currently in the OECD Test Guideline Programme (OECD TGP) work plan (EURL-ECVAM 2019). Furthermore, the European Food Safety Authority (EFSA) recommends the use of the in vitro comet assay in combination with specific enzymes to detect oxidized bases and also to provide complementary information of the genotoxic mechanisms of action of nanomaterials (EFSA 2018).

In fact, the standard alkaline comet assay only detects SBs and AP-sites, whereas most DNA-damaging agents induce other lesions such as oxidized and alkylated bases, adducts or cross-links. To partly overcome this limitation, the comet assay has been modified including a digestion step after lysis with specific DNA-repair enzymes (DNA glycosylases). These enzymes can detect and remove the damaged base, leaving an AP-site, which is then converted to a SB by an associated AP lyase activity of the enzyme or, if the enzyme lacks this activity, by the alkaline pH of the unwinding solution (Azqueta and Collins 2013; Muruzabal et al. 2020a). The frequency of the net enzyme-sensitive sites is calculated by subtracting the DNA damage level of the nucleoids incubated with the enzyme buffer alone from the DNA damage level of the nucleoids treated with the enzymes (Collins 2009). Most commonly, this modified version of the comet assay has been applied for the detection of oxidized bases using formamidopyrimidine DNA glycosylase (Fpg), endonuclease III (Endo III) and human 8-oxoguanine DNA glycosylase I (hOGG1) (Olsen et al. 2003; Smith et al. 2006; Hansen et al. 2010; Collins 2014; Muruzabal et al. 2020a). The use of enzymes for the detection of alkylated DNA lesions, such as 3-methyladenine DNA glycosylase II (AlkA), 3-methyladenine DNA glycosylase (AlkD), and human alkyladenine DNA glycosylase (hAAG), has also been reported (Collins et al. 2001; Hašplová et al. 2012; Muruzabal et al. 2020b). We recently published a review of the enzymes that have been used in combination with the comet assay that identified 12 different enzymes used for detecting several lesions, mainly oxidized bases (both purines and pyrimidines), but also alkylated bases, uracil residue and pyrimidine dimers (Muruzabal et al. 2020a).

Similarly, although less extensively, the comet assay has also been modified for the detection of cross-links. When DNA contains inter-strand cross-links (ICL) within its structure, the extension during electrophoresis in the comet assay is inhibited as the nucleoid is kept compact, thereby exhibiting the opposite effect compared to SBs; migrating less compared to DNA of control cells. Thus, ICLs can be detected either by increasing the duration of the electrophoresis to such an extent that even DNA of non-treated cells migrates considerably, or by treating cells with a second genotoxic agent (chemical or physical) for inducing a known level of DNA breaks. DNA containing ICL will migrate less compared to DNA of control cells or to the cells treated with the second genotoxic agent depending on the approach used (Olive et al. 1992; Tice et al. 1997, 2000).

The objective of this work is to internally validate the performance of two modified versions of the comet assay for the detection of different genotoxic endpoints. Particularly, four different commercially available enzymes (hAAG, hOGG1, Fpg, and Endo III), and a widely used non-commercial crude bacterial extract of Fpg, have been used in combination with the comet assay for the analysis of the DNA lesions induced by compounds with different mechanisms of action (i.e., oxidizing and alkylating agents) to detect several lesions within a single assay. Additionally, cells treated with these compounds were also analyzed employing the comet assay modified for the detection of cross-links. Moreover, the activity of the different enzymes employed in this work and their specificity towards a set of different defined DNA lesions were determined using a multiplex oligonucleotide-cleavage assay (the Glyco-SPOT assay). The compounds employed were potassium bromate (KBrO_3_, oxidizing compound mainly inducing 8-oxoguanines), methyl methanesulfonate (MMS, monofunctional alkylating agent), ethyl methanesulfonate (EMS, monofunctional alkylating agent), hydrogen peroxide (H_2_O_2_, oxidizing compound), cisplatin (CisPt, cross-linking agent), mitomycin C (Mit. C, cross-linking agent), benzo[a]pyrene diol epoxide (BPDE, bulky-adduct inducer), dimethyl sulfoxide (DMSO, as non-genotoxic control), and Triton X-100 (as cytotoxic compound). The final aim of this work was to provide an assay that could be used as a tool to generate mechanistic information in the current regulatory context for chemical risk assessment.

## Material and methods

### Chemicals and reagents

Low melting point agarose, standard agarose, Triton X-100, Tris base, HEPES, Na_2_EDTA, bovine serum albumin (BSA), NaOH, KCl, KBrO_3_, MMS, EMS, H_2_O_2_, CisPt, Mit. C, and 4’,6-diamidino-2-phenylindole (DAPI) were purchased from Sigma–Aldrich. BPDE was purchased from Santa Cruz Biotechnology. DPBS 1 × for mixing cell suspensions with agarose was purchased from Gibco. DPBS without Ca^+2^ and Mg^+2^ 10 × from Lonza was used to prepare PBS 1 × washing solutions for comet assay slides. DMSO was purchased from PanReac AppliChem. All cell culture reagents were purchased from Gibco.

The enzymes hAAG, Endo III, and commercial Fpg were purchased from New England Biolabs (catalog number M0313S, M0268S, and M0240S, respectively); hOGG1 was purchased from R&D Systems, Bio-Techne (catalog number 4130-100-EB). Non-commercial Fpg (Fpg.A) from an over-producing *E. coli* strain was kindly provided by NorGenoTech AS (Oslo, Norway) that distributes the enzyme on request.

### Cell culture

Human-derived lymphoblastoid TK-6 cell line was obtained from the American Type Culture Collection (ATCC). Cells were grown in RPMI medium (Roswell Park Memorial Institute; ref. A10491-01, Gibco) containing D-glucose, HEPES, L-glutamine, sodium bicarbonate, and sodium pyruvate and supplemented with 10% heat-inactivated fetal bovine serum, 100 U/mL penicillin and 0.1 mg/mL streptomycin. Cells were maintained as a suspension culture, between 0.2 and 1 × 10^6^ cells/mL, in continuous agitation at 37 °C in a humidified atmosphere with 5% CO_2_. Cells in a passage number lower than 20, were maintained in culture for no longer than 2 months since thawed.

### Treatment of cells

Table 1 shows the different compounds and concentrations tested, including the solvent used and the relative suspension growth (RSG) for each compound. Preliminary studies were performed to assess cytotoxicity employing the proliferation assay according to Azqueta et al. (2013a) with some modifications. Briefly, cell suspensions were counted before and just after treatment and after 24 and 48 h in fresh culture. The criterion for cytotoxicity was the RSG at 48 h. For that purpose, total suspension growth (TSG) was calculated for each condition dividing the number of cells after 48 h by the number of cells treated. Counting was carried out using trypan blue. The RSG was calculated by dividing the TSG from each condition tested by the TSG of the solvent control. Three concentrations, being the highest around 80% RSG were selected for the enzyme-modified comet assay experiments. An additional concentration of each compound with RSG values below 80% was employed for the comet assay modified for the detection of cross-links (two additional concentrations in the case of CisPt) except for H_2_O_2_, which was not tested using this modification (Table 1). Triton X-100 was employed as a cytotoxicity control; thus its RSG values were lower: 60% for the lowest concentration (0.03 mM) and RSG < 10% for the highest (0.1 mM).

**Table 1:** List of compounds, CAS numbers, solvents, concentrations employed, and their respective RSG (%)

Compound	CAS number	Solvent*	Concentrations**	RSG
KBrO_3_	7758-01-2	H_2_O	0.313 mM	90
			0.625 mM	85
			1.25 mM	78
			2.5 mM	40
MMS	66-27-3	DMSO	5 µM	98
			10 µM	85
			20 µM	80
			40 µM	58
EMS	62-50-0	DMSO	0.5 µM	90
			5 µM	84
			50 µM	77
			100 µM	40
BPDE	55097-80-8	DMSO	0.025 µM	93
			0.05 µM	83
			0.1 µM	70
			0.2 µM	50
H_2_O_2_	7722-84-1	PBS	125 µM	92
			250 µM	87
			500 µM	79
CisPt	15663-27-1	H_2_O	0.83 µM	100
			1.66 µM	100
			3.33 µM	80
			6.66 µM	39
			13.33 µM	12
Mit. C	50-07-7	Medium	0.006 µM	98
			0.03 µM	95
			0.15 µM	82
			0.3 µM	50
Triton X-100	9002-93-1	H_2_O	0.03 mM	60
			0.1 mM	7
DMSO	67-68-5	Medium	1%	100
			2%	95
			4%	83
			8%	80

*The final solvent concentration was 1% in all cases. **The three lowest concentrations of each compound were employed for the enzyme-modified comet assay. The same three concentrations and the highest one (the two highest in the case of CisPt) of each compound were used for the comet assay modified for ICL detection (except for H_2_O_2_ and Triton X-100)

Treatments were performed as follows: TK-6 cells were seeded in a 12-well plate (1 mL/well) at 1 × 10^6^ cells/mL in culture medium containing no serum and treated for 3 h with different non-cytotoxic concentrations of the compounds or their vehicles. Treatments were performed with continuous agitation at 37 °C in a humidified atmosphere with 5% CO_2_. In the case of H_2_O_2_ the treatment was performed in phosphate-buffered saline (PBS) at 4 °C for 5 min, and cells were then washed and seeded in complete culture medium for 1 h and 45 min to repair the strand breaks and leave only the oxidized lesions (these conditions were used based on preliminary studies, data not shown).

After 3 h of treatment, cells were kept ice-cold to prevent DNA repair and were centrifuged and washed twice with PBS. After the second centrifugation cells were resuspended in PBS to a final concentration of 2.5 × 10^5^ cells/mL. These cells were directly employed for the comet assay (see next section). Each compound was tested three times in the same conditions.

### Enzyme-modified comet assay

Recently published recommendations for describing comet assay procedures and results were followed (Møller et al. 2020).

The medium-throughput format of 12 minigels per slide of the comet assay (Shaposhnikov et al. 2010) was employed according to Muruzabal et al. (2018).

The enzyme hAAG and the non-commercial Fpg were previously titrated (Muruzabal et al. 2018, 2020b) using MMS and KBrO_3_ to induce alkylated and oxidized bases, respectively. In the case of commercial Fpg and hOGG1, they were titrated in preliminary studies using KBrO_3_ to induce oxidized purines. For the titration of Endo III, oxidized pyrimidines were induced by H_2_O_2_ (cells were incubated under the above mentioned culture conditions for SB repair after treatment for 1 h 45 min). The same reaction buffer (see below) was used for all the enzymes. Based on these experiments the selected enzyme concentrations were 0.026, 10, 6.66 and 33.3 U/mL for commercial Fpg, hAAG, hOGG1, and Endo III, respectively. Regarding the non-commercial Fpg (Fpg.A), as it was not possible to determine its activity in terms of U/mL, a dilution 1:300,000 from the original stock in the enzyme-reaction buffer was used.

To set the minigels, 30 µL of cell suspension (either treated or non-treated cells, previous section) were mixed with 140 µL of 1% low melting point agarose in PBS at 37 °C. Then, 5 µL droplets of the corresponding cell suspension-agarose mix were placed on agarose-precoated slides. The slides were placed on the metal holder of the 12-Gel Comet Assay Unit™ (NorGenoTech, Oslo, Norway), which contains a template to set the minigels in precise positions (2 rows of 6) and was previously cooled in the fridge. Each slide contained 12 minigels of the same suspension, so 6 replicates were designed for incubation: one pair of minigels to be incubated with the enzyme-reaction buffer and the remaining five pairs to be incubated with each of the enzymes. Therefore, as three concentrations and control cells (i.e*.*, cells exposed to the compound solvent) were tested with each compound, four slides per compound were prepared.

Once slides were prepared, cells were lysed by immersing the slides in lysis solution (2.5 M NaCl, 0.1 M Na_2_EDTA, 10 mM Tris base, pH 10, and 1% Triton X-100) at 4 °C for 1 h. Then slides were washed three times, 5 min each, at 4 °C with the reaction buffer of the enzymes (40 mM HEPES, 0.1 M KCl, 0.5 mM Na_2_EDTA, 0.2 mg/ml BSA, pH 8). During the washes, the enzymes were prepared by diluting original stocks with reaction buffer. It should be noted that the enzyme-reaction buffer employed for the washes was also employed for diluting all enzymes to their optimal concentration.

After washing, slides were transferred to a cold 12-Gel Comet Assay Unit™ to incubate each of the gels separately, as gels are isolated in wells on the slides. Units were placed on a cold metal plate to keep them cold during the enzymes or reaction buffer addition to avoid enzymatic reactions until incubation. Then, 30 µL of reaction enzyme buffer or of the corresponding enzyme were pipetted to each well and a clean slide was placed on top of the unit to cover all wells and prevent contamination and evaporation. The 12-Gel Comet Assay Units™ were transferred to a pre-heated moist box and placed in the incubator for 1 h at 37 °C.

After incubation, units were placed on a cold plate to stop the enzyme-reaction and slides were removed from the chambers and immediately transferred to the electrophoresis tank for unwinding in electrophoresis solution (0.3 M NaOH, 1 mM Na_2_EDTA, pH > 13) for 40 min in a 4 °C cold room. Afterwards, electrophoresis was carried out at 1.2 V/cm for 20 min at 4 °C.

After electrophoresis, slides were neutralized by washing them in PBS for 10 min and rinsed in distilled water for further 10 min. To dehydrate the gels for avoiding edge effect (i.e., comets going in different angles) (Azqueta et al. 2013b), slides were immersed in 70% ethanol for 15 min and then in absolute ethanol for further 15 min and left to dry overnight at room temperature.

Finally, each minigel was stained with a 5 µL drop of 1 µg/mL of DAPI and all minigels of the slide were covered using a coverslip (24 × 60 mm). After 30 min of incubation with DAPI at room temperature, slides were analyzed using the semi-automated image analysis system Comet Assay IV (Instem Perceptive Instruments) and 50 nuclei per gel, 100 per condition, were scored. The percentage of DNA in tail (or tail intensity) was used as descriptor for each comet. The median percentage of DNA in tail for 50 comets was calculated for each of the duplicate minigels and the mean of the two medians was taken as the measure of DNA damage for each condition in each of the three independent experiments. Net enzyme-sensitive sites were calculated by subtracting the percentage tail DNA obtained with the buffer incubation alone from that obtained after incubation with each enzyme. The value of buffer incubation alone was representative of SB and ALS of each sample. Three independent experiments were carried out and results are expressed as the mean of the three experiments ± SD.

### Modification of the comet assay to detect cross-links

The format of 12 minigels per slide of the comet assay was also employed for this modification. Procedure for setting the gels was the same as previously described. However, prior to the lysis step slides were transferred to the 12-Gel Comet Assay Units™ for treating the gels with H_2_O_2_ to induce a known amount of DNA damage (i.e., around 40–50% of DNA in tail, in terms of tail intensity). Particularly, gels were treated for 5 min at ice-cold temperature with H_2_O_2_ 98 mM (100 µL per well) (concentration and conditions established according to preliminary experiments) and then washed with cold PBS. Then slides were placed in a Coplin jar for the lysis step. From this point, remaining procedure was the same as in the previous section, but no enzyme or reaction buffer incubation step was performed with these slides (i.e., slides were immersed in the electrophoresis solution for the alkaline treatment after the lysis). All solutions, reagents and processing of the results were the same as explained in  "Enzyme-modified comet assay" section.

### Glyco-SPOT assay

The multiplexed oligonucleotide (ODN) cleavage assay on support (i.e., Glyco-SPOT assay, LXRepair, La Tronche, France) was used according to Muruzabal et al. (2020b) to simultaneously control the activity of the enzymes toward several potential substrate lesions. In brief, the assay consists of 24-well glass slides (Streptavidin-coated, XanTec bioanalytics, Germany) functionalized with a panel of ODNs bearing different lesions. Particularly, the included lesions were: 8-oxoguanine paired with cytosine (8oxoG–C), adenine paired with 8oxoG (A–8oxoG), ethenoadenine paired with thymine (EthA–T), hypoxanthine paired with thymine (Hx–T), tetrahydrofuran (which is an abasic site stable analog) paired with adenine (THF–A), thymine glycol paired with adenine (Tg–A) and uracil paired either with guanine or with adenine (U–G and U–A, respectively). All lesions were labeled with a Cy3 at their end as described in Candéias et al. (2010) and Pons et al. (2010).

Each ODN was immobilized in duplicate in each well together with a Control-ODN that contained no modification. As hAAG was previously analyzed using this assay (Muruzabal et al. 2020b), the procedure was only performed with both Fpgs, hOGG1 and with Endo III. Particularly, five concentrations of each enzyme were tested (Fpg: 0.0002, 0.001, 0.005, 0.01, and 0.05 U/well; Fpg.A: 1:2,000,000, 1:1,000,000, 1:500,000, 1:100,000, and 1:20,000 dilutions from the original stock; hOGG1: 0.00016, 0.0008, 0.004, 0.02, and 0.1 U/well; Endo III: 0.0008, 0.004, 0.02, 0.1, and 0.5 U/well) in two different wells in the same enzyme-reaction buffer employed for the enzyme-modified comet assay (see "Enzyme-modified comet assay" section for details). Each well contained 80 µL of buffer. The excision reaction was run for 60 min at 37 °C under agitation (700 rpm). Then the slides were washed 2 × 5 min in PBS containing 0.2 M NaCl—0.05% Tween^®^20 and dried by centrifugation.

The fluorescence of each spot was quantified at 532 nm wavelength using the Innoscan 710AL scanner from Innopsys (Toulouse, France) and the associated MAPIX software. Data were normalized as described using NormalizeIt software (Millau et al. 2008). To calculate the final cleavage rate of each ODN-containing lesion, the fluorescence of the control well, incubated with the enzyme-reaction buffer only, was taken as reference (100% fluorescence). The data were also corrected by the control-ODN cleavage rate that remained below 10%. Finally, the lesion-ODN cleavage percentage was 100 × (1 − percentage of fluorescence of lesion-ODN/percentage of fluorescence of control-ODN).

### Statistical analysis

Non-parametric one-way analysis of variance (i.e., Kruskal–Wallis test) followed by Bonferroni test was applied to compare the levels of net enzyme-sensitive sites (in terms of % DNA in tail), in the enzyme-modified comet assay, and the levels of SBs, in the assay modified for the detection of cross-links, obtained in TK-6 cells treated with different compounds with their respective controls. Differences showing *p* value < 0.05 were considered statistically significant. Analyses were carried out using STATA v.12.0 software package.

## Results

In preliminary experiments, we measured RSG using the proliferation assay with a broad range of concentrations of each chemical to perform the final assays with non-cytotoxic concentrations (RSG 48 h after the treatment > 80%) and including a cytotoxic concentration (for the comet assay modified for ICL detection an additional concentration with RSG < 80%) (Table 1).

### Enzyme-modified comet assay

**Fig. 1:**
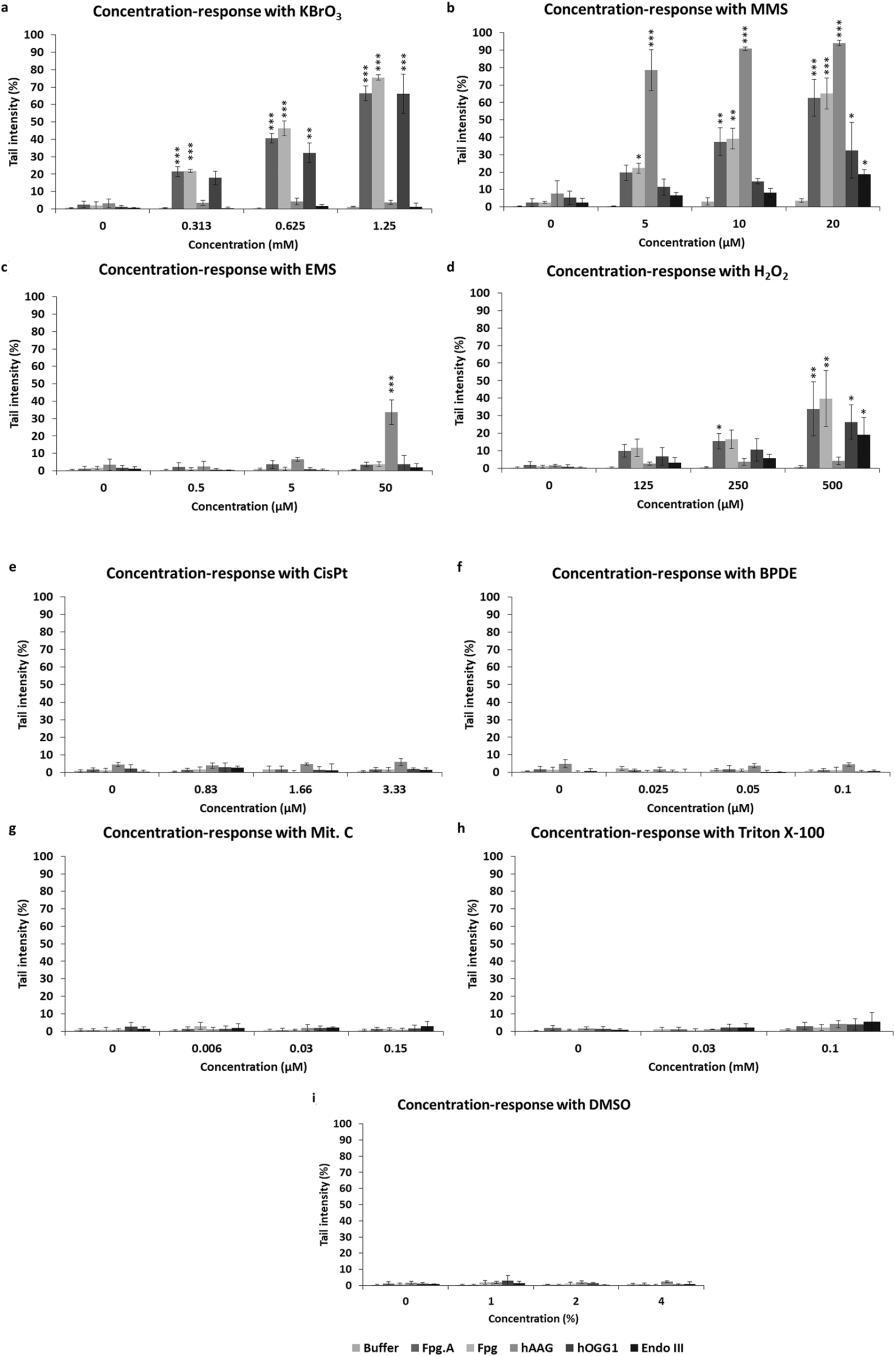
Enzyme-modified comet assay. Results (tail intensity) obtained in TK-6 cells after 3 h of treatment with different compounds (**a**–**i**) are expressed as SB and ALS (in “Buffer”) or net enzyme-sensitive sites (in the enzymes) ± SD (*n* = 3 independent experiments). Fpg. A: non-commercial Fpg. *p < 0.05, **p < 0.01, ***p < 0.001, significantly different from control cells

Figure 1 shows the results from the nucleoids treated with the enzyme buffer and the net enzyme-sensitive sites of each enzyme. In all conditions tested (i.e., treated, and non-treated cells) with all compounds, the level of DNA damage (in terms of % tail intensity) obtained with the enzyme buffer (“Buffer”) was below 5% (Fig. 1a–i), indicating that very low levels of SBs were induced at concentrations employed with the tested chemicals. Furthermore, after analyzing “Buffer” values of each compound, no concentration-related effects were observed.

The cells treated with KBrO_3_ (Fig. 1a), an oxidizing compound (induces mainly 8-oxoguanines), showed a very similar response between non-commercial and commercial Fpg enzymes (“Fpg.A” and “Fpg”, respectively). A highly significant increase in enzyme-sensitive sites for both versions of Fpg, KBrO_3_ concentration dependent, was found compared to non-treated cells. Indeed, highly significant differences (*p* < 0.001) were found in all KBrO_3_ concentrations tested compared to non-treated cells (Fig. 1a). hOGG1 response was similar to the one obtained with both Fpg enzymes, but the levels of hOGG1-sensitive sites were slightly lower at the lowest KBrO_3_ concentrations. When comparing hOGG1-sensitive sites in treated versus non-treated cells, significant differences were found from 0.625 mM of KBrO_3_ onwards. hAAG and Endo III did not show any activity in KBrO_3_-treated cells, and no increase in hAAG- or Endo III-sensitive sites was detected compared to non-treated cells at any of the tested concentrations.

MMS, a monofunctional alkylating agent, also induced a similar response in treated cells when analyzed with both Fpgs (Fig. 1b). A concentration-dependent increase in enzyme-sensitive sites was observed with both Fpg enzymes, being significant from 5 µM onwards in the case of commercial Fpg, and from 10 µM onwards in the case of non-commercial Fpg. Regarding hAAG, the increase in enzyme-sensitive sites in treated cells was highly significant (*p* < 0.001) compared to non-treated cells from the lowest concentration onwards, and more sharpened compared to both Fpgs. The response observed with hOGG1 and Endo III also showed an MMS concentration-dependent increase, but in a more limited way, as the enzyme-sensitive sites were considerably lower compared to the other enzymes; significant differences (*p* < 0.05) compared to non-treated cells were only found at 20 µM MMS (Fig. 1b).

EMS is another monofunctional alkylating agent inducing ethyl groups, and its effects on treated cells (in terms of enzyme-sensitive sites) were only noticeable at the highest concentration (i.e., 50 µM) with hAAG (Fig. 1c). Indeed, the level of hAAG-sensitive sites in cells treated with 50 µM of EMS was significantly higher compared to levels detected in non-treated cells (*p* < 0.001). Regarding the other enzymes tested, no activity was detected at any of EMS concentrations tested (Fig. 1c).

H_2_O_2_ induces both oxidized DNA lesions and SB in DNA of treated cells. To study base damage specifically, cells were incubated after treatment to repair SB leaving only oxidized DNA damage. Both Fpgs enzymes showed a similar pattern in detecting H_2_O_2_-induced lesions in TK-6 cells, showing a concentration-dependent increase in enzyme-sensitive sites (Fig. 1d). Indeed, significant differences compared to non-treated cells were found at 250 and 500 µM H_2_O_2_ with Fpg.A (*p* < 0.05 and *p* < 0.01, respectively) and at 500 µM with Fpg (*p* < 0.01). The pattern of response observed with hOGG1 was similar to the observed with both Fpgs but with lower tail intensities. A significant increase in DNA damage was only detected at the highest H_2_O_2_ concentration (i.e., 500 µM) compared to non-treated cells (*p* < 0.05). Regarding Endo III, a small but significant increase in enzyme-sensitive sites was found at the highest concentration of H_2_O_2_ (500 µM) (*p* < 0.05). No response was observed with hAAG at any of H_2_O_2_ concentrations tested (Fig. 1d).

No activity was detected for any enzyme at any of the tested concentrations of CisPt (cross-linking agent) (Fig. 1e), BPDE (bulky adducts inducer) (Fig. 1f), and Mit. C (cross-linking agent) (Fig. 1g). Finally, regarding Triton X-100 and DMSO, the non-genotoxic compounds employed, no increase in DNA damage was detected at any of the tested concentrations with any of the enzymes (Fig. 1h, i, respectively). Overall, no statistically significant differences were found between the two Fpgs with any of the tested compounds.

### Modification of the comet assay for the detection of cross-links

Compared to the enzyme-modified comet assay, an additional higher concentration of each compound with RSG levels < 80% was tested. For the detection of cross-links, in addition to the treatment with each compound, a second treatment was performed (once comet assay gels were molded on the slides) with H_2_O_2_ to induce about 40–50% of DNA damage (in terms of tail intensity).

**Fig. 2:**
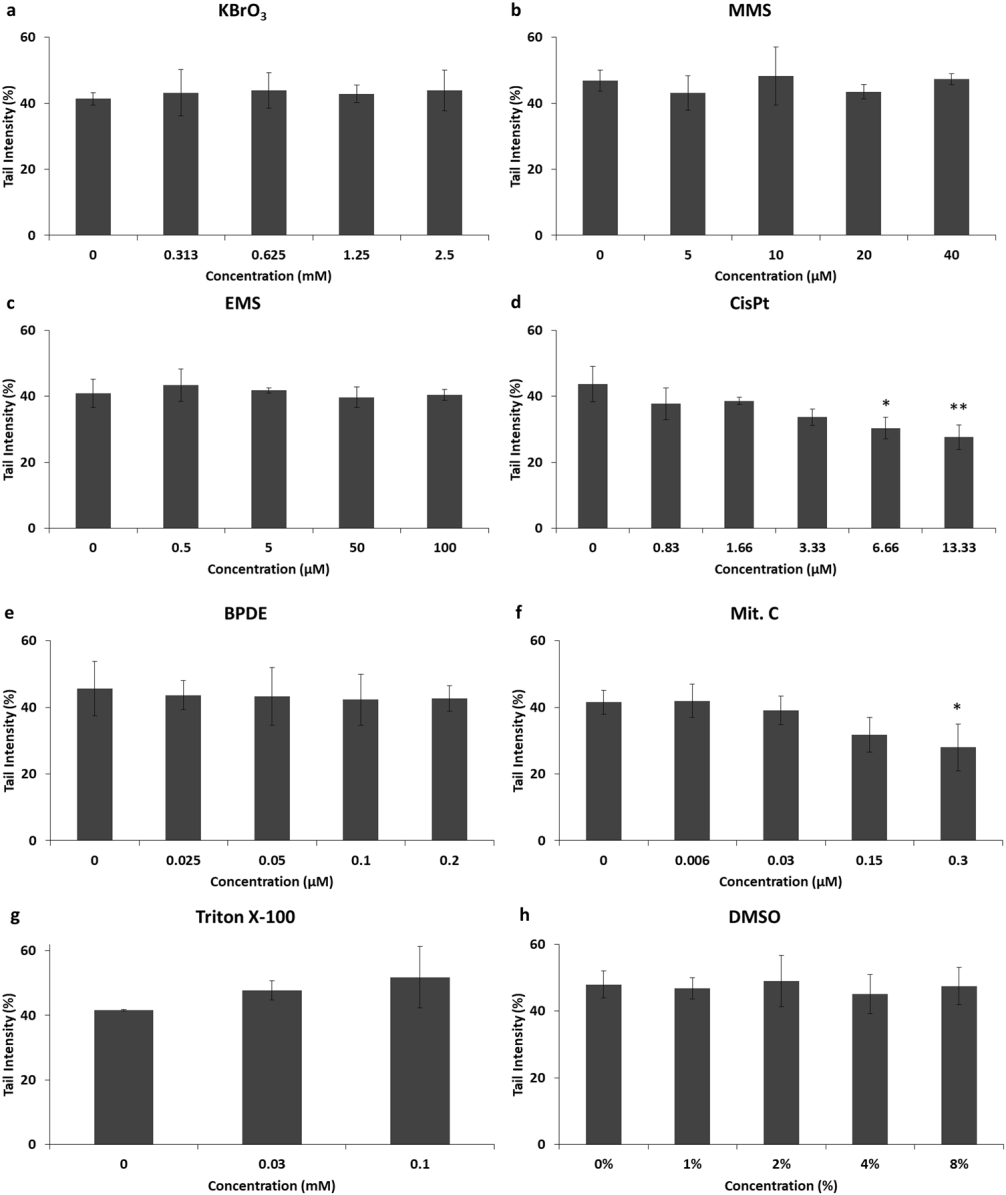
Modification of the comet assay for cross-links detection. Figures show results (tail intensity) obtained with TK-6 cells treated for 3 h with different compounds (**a**–**h**) and treated again (once set for the comet assay) with H_2_O_2_ to induce around 40–50% of DNA in tail (i.e., DNA damage). Mean of SB ± SD (*n* = 3 independent experiments) are represented. Reduction of tail migration in terms of tail intensity indicates the presence of cross-links. *p < 0.05, **p < 0.01, significantly different from control cells

In cells treated with CisPt, a concentration-dependent decrease in the levels of H_2_O_2_-induced DNA damage was observed, with 43 ± 5.3% of DNA in tail in non-treated cells and 27 ± 3.7% of DNA in tail at the highest CisPt concentration, representing a reduction of 35 ± 15.5% of DNA migration (Fig. 2d). Indeed, this reduction was significant (*p* < 0.05) and highly significant (*p* < 0.01) at 6.66 and 13.33 µM of CisPt, respectively (Fig. 2d). Mit. C also induced a concentration-dependent decrease in H_2_O_2_-induced DNA damage (Fig. 2f). This reduction was especially pronounced at the highest Mit. C concentrations tested, at which tail intensity levels were reduced from 41.5 ± 3.5% in non-treated cells to 31 ± 5.2% and 27 ± 7% in 0.15 and 0.3 µM, respectively, which represent reductions of DNA migration of 23 ± 12.2% and 33 ± 13.3%, respectively. In the case of the highest concentration (i.e., 0.3 µM) of Mit. C, the reduction in detected DNA damage was significant compared to non-treated cells (*p* < 0.05).

Regarding cells treated with the other genotoxic compounds (KBrO_3_, MMS, EMS, and BPDE) (Fig. 2a–c, e) the H_2_O_2_-induced DNA damage did not show either an increase or a decrease with any of the compounds tested. Indeed, DNA damage values (in terms of tail intensity) found with all concentrations in all compounds remained at 40–50% of DNA in tail with no significant variations (Fig. 2a–c, e). Similarly, cells treated with non-genotoxic compounds (Triton X-100 and DMSO) (Fig. 2g, h) did not show any statistically significant variation in detected DNA damage, which in all cases was 40–50%. However, Triton X-100 seemed to induce a slight concentration-dependent increase in DNA damage, although it was not significant compared to control cells (Fig. 2h).

### Glyco-SPOT assay

**Fig. 3:**
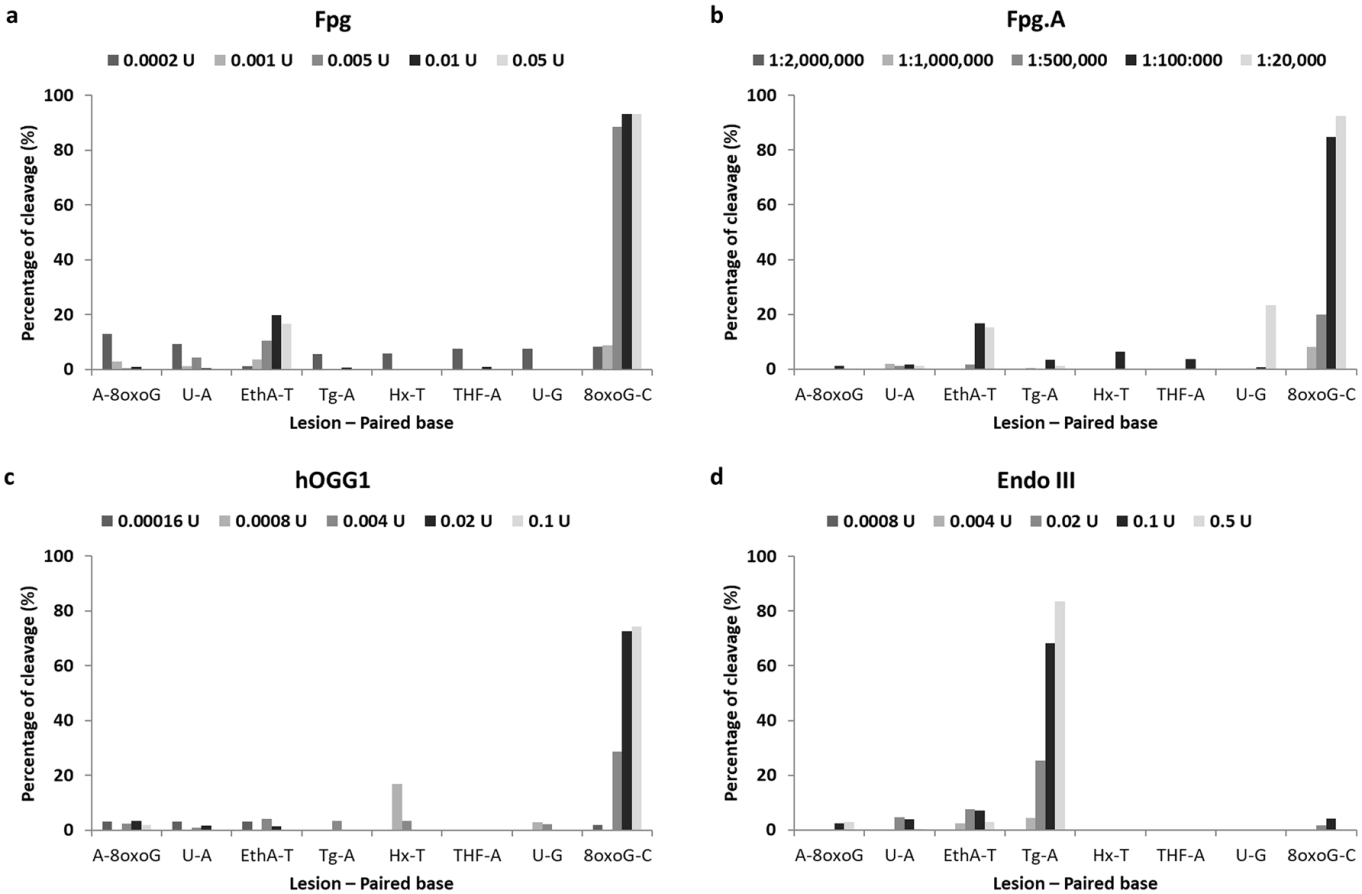
Normalized percentage of cleavage induced by the enzymes in the different DNA lesions included in the Glyco-SPOT assay. Figures show results obtained with commercial Fpg (**a**), non-commercial Fpg (Fpg.A) (**b**), hOGG1 (**c**), and Endo III (**d**). A–8oxoG: A paired with 8oxoG; 8oxoG–C: 8oxoG paired with C; Hx–T: hypoxanthine paired with T; EthA–T: ethenoadenine paired with T; Tg–A: thymine glycol paired with A; THF–A: tetrahydrofuran—abasic site stable analog—paired with A; U–G: uracil paired with G; and U–A: uracil paired with A

Incubation of the different lesions with increasing concentrations of Fpg, Fpg.A and hOGG1 resulted essentially in the cleavage of 8-oxoguanine paired with cytosine (8oxoG–C) (Fig. 3a–c), while Endo III cleaved thymine glycol paired with adenine (Tg–A) (Fig. 3d). Results regarding hAAG are published elsewhere; it cleaved hypoxanthine and ethenoadenine, and did not detect oxidized bases (Muruzabal et al. 2020b).

## Discussion

The study of mechanisms of action and its potential linkage with AO is gaining relevance in toxicological evaluation in a regulatory context. For this reason, developing tools for the detection of the KE involved in AOPs becomes essential for the development of this mechanistic approach for risk assessment. The inclusion of an in vitro comet assay with its mechanistic modifications is in line with the strategic inclusion of so-called New Approach Methodologies (NAMs) in human risk assessment (Parish et al. 2020).

In this study, we evaluated the performance of two modifications of the comet assay for detecting an extended variety of DNA lesions induced by compounds with different mechanisms of action. Regarding the use of enzymes, we employed different enzymes for the detection of oxidized bases (hOGG1 and Fpg for oxidized purines and Endo III for oxidized pyrimidines) as well as alkylated bases (using hAAG). We also included a non-commercial Fpg preparation (from an over-producing *E. coli* strain) (Fpg.A), which is widely employed among comet assay users, to compare its performance with a commercial Fpg.

As previously explained, when performing the enzyme-modified comet assay, all compounds were evaluated at non-cytotoxic concentrations (RSG > 80%, Table 1) to reduce the chance of false positive results, as toxicity may induce DNA damage detected in the Comet assay (Henderson et al. 1998). Moreover, we included Triton X-100 as non-genotoxic but cytotoxic compound, at medium (60% RSG) and high cytotoxic (RSG < 10%) concentrations. Interestingly, under our conditions negative results would be obtained with the standard comet assay with the genotoxic compounds, as results obtained with the enzyme buffer alone did not show DNA damage, which suggest that no SBs were induced under the conditions tested. This indicates that the enzyme-modified comet assay significantly increases not only the sensitivity of the assay but also its specificity, providing mechanistic information about the type of damage.

To date, Fpg is one of the most used DNA-repair enzymes in combination with the enzyme-modified comet assay (Muruzabal et al. 2020a). Fpg is a bacterial enzyme able to detect 8-oxoguanine, other purine oxidation products, and ring-opened purines (also known as formamidopyrimidines—Fapy—). For this reason, Fpg also detects N7-guanine adducts indirectly formed from alkylated bases, as these lesions turn into Fapy due to the high pH of the lysis solution in the comet assay (Speit et al. 2004; Hansen et al. 2018; Muruzabal et al. 2020b). We compared the performance of the commercial Fpg and the non-commercial bacterial Fpg extract (from an over-producing *E. coli* strain), both of them widely used in the comet assay. No relevant differences were observed between both enzymes towards different levels of DNA lesions induced by several compounds (Fig. 1a-i). The highest level of enzyme-sensitive sites was found with KBrO_3_ (Fig. 1a), which specifically induces oxidized purines (mainly 8-oxoguanines) with little or no induction of SBs (Møller et al. 2015), and with MMS (Fig. 1b), a monofunctional alkylating agent. As previously reported, results with both compounds were as expected, in the case of MMS due to the aforementioned conversion of N7-alkylated adducts into Fapy, which are detectable by Fpg (Speit et al. 2004; Hansen et al. 2018; Muruzabal et al. 2020b). The treatment with H_2_O_2_ also induced Fpg-sensitive sites (Fig. 1d), although to a much lesser extent compared to KBrO_3_ and MMS. For the other compounds tested, no increases in DNA damage levels were detected by the Fpgs under our testing conditions. When we analyzed the specificity for DNA lesions of the Fpg using the Glyco-SPOT assay (Fig. 3a, b), similar response was obtained, as cleavage was only detected for 8-oxoguanine (paired with cytosine) (Fig. 3c).

hOGG1 is the human functional homologue of Fpg. This glycosylase initiates excision of oxidized purines (mainly 8-oxoguanine paired with cytosine) (Bjorâs et al. 1997; Radicella et al. 1997), although it has been reported that is also able to detect Fapy-guanines (Bjorâs et al. 1997; David and Williams, 1998; Lukina et al. 2013). The DNA lesion levels detected using hOGG1 with the oxidizing agents, KBrO_3_ and H_2_O_2_, were very similar to that observed with Fpg, but the levels of hOGG1-sensitive sites were lower in the case of H_2_O_2_ (Fig. 1a, d). Additionally, hOGG1-sensitive sites were detected in MMS-treated cells, mainly at the highest concentration tested (Fig. 1b). This is coherent with its ability to detect Fapy-guanines (Bjorâs et al. 1997; David and Williams 1998; Lukina et al. 2013). One plausible explanation is that, under our conditions, the hOGG1 affinity for Fapy lesions (derived from the transformation of alkylated bases to ring-opened purines due to the alkaline conditions of the comet assay) is lower than that of Fpg, as the detected levels with hOGG1 were considerably lower. Alternatively, we can also hypothesize that at the highest concentration used, MMS may be inducing an oxidative stress to the cells, which can cause the oxidation of bases as a secondary mechanism of action (Mizumoto et al. 1993). As expected, hOGG1 did not show any activity with the other compounds tested, either genotoxic or non-genotoxic. Regarding Glyco-SPOT assay for evaluating hOGG1 specificity, substrate specificity was similar as with Fpg enzymes, (8-oxoguanine paired with cytosine (Fig. 3c).

Endo III is a bacterial glycosylase involved in the excision of a wide range of oxidized pyrimidines, including thymine glycol, 5-hydroxycytosine or cytosine glycol (Doetsch and Cunningham 1990; David and Williams 1998). Endo III-sensitive sites were detected in cells treated with H_2_O_2_ and MMS. Results obtained with H_2_O_2_, although expected and significantly higher compared to non-treated cells, were low in terms of enzyme-sensitive sites when compared with hOGG1 or Fpg (Fig. 1d). A plausible explanation is that oxidized lesions induced by H_2_O_2_ may be preferentially located on purine bases, as guanine is the most easily oxidizable DNA base (Cadet et al. 2014). As with hOGG1, although in a lesser extent, Endo III-sensitive sites were detected in cells exposed to MMS, especially at the highest concentrations (Fig. 1b). It has been reported that Endo III can detect Fapy adenines (Dizdaroglu et al. 2000), but as methylation of adenine induced by MMS occurs mainly at the N3 of the base (Beranek 1990) and thereby not occurring in the imidazole ring of the purine, this modification cannot be converted into ring-opened purine. For such reasons, and as aforementioned with hOGG1, the most plausible explanation is that high MMS concentrations may be inducing an oxidative stress, which ultimately can cause the oxidation of bases as a secondary mechanism of action. Interestingly, no Endo III-sensitive DNA lesions were observed with KBrO_3_, indicating that no oxidized purines were detected with Endo III. Finally, no response was observed with other genotoxic and non-genotoxic compounds tested. As expected, Endo III only showed specific activity towards thymine glycol paired with adenine in the Glyco-SPOT assay (Fig. 3d).

Smith et al. (2006) compared the substrate specificity of Fpg, Endo III, and hOGG1 in the enzyme-modified comet assay using KBrO_3_ (to induce DNA oxidation) and MMS (to induce DNA alkylation) in mouse lymphoma cells. In a different cell line, their results with KBrO_3_ when using Fpg and hOGG1 were largely similar to ours. However, they detected KBrO_3_-derived lesions with Endo III, especially at the highest concentration tested with a RSG of 39%, which is a level of toxicity higher than in our study. Regarding results with MMS, they also observed a high response with Fpg at all concentrations tested. Interestingly, they observed a concentration-dependent increase in Endo III-sensitive sites in MMS-treated cells in a much greater extent compared to our results. As they also observed Endo III-sensitive sites after treating the cells with KBrO_3_, we speculate that these differences may be due to the differences in cellular response to the genotoxic agent in the two cell lines. Moreover, the use of a non-commercial enzyme batch of Endo III (crude bacteria extract) by these authors may also underlie the differences between both studies.

Unlike our results, Smith and colleagues (2006) did not detect hOGG1-sensitive sites in MMS-treated cells, whereas we measured significant increases in hOGG1-sensitive sites at the highest MMS concentration (i.e., 20 µM) for which RSG value was 80%, as compared to the highest concentration tested in their study (i.e., 22.7 µM) with RSG value of 92%. This difference in cytotoxicity combined with the fact that different cell lines are being employed may partially explain these differences.

Recently, we described the use of hAAG in combination with the comet assay for the first time (Muruzabal et al. 2020b). This enzyme is a monofunctional glycosylase responsible for initiating the base excision repair (BER) pathway for repairing alkylated bases. Particularly, the enzyme detects 3-methyladenine and 7-methylguanine (O’Connor 1993), 1-methylguanine (Lee et al. 2009) as well as other non-alkylated lesions including deaminated purine lesions (i.e*.*, hypoxanthine and xanthine) and the lipid peroxidation-derived adduct 1,*N*6-ethenoadenine (Lee et al. 2009; Taylor et al. 2018). Moreover, it has been hypothesized that hAAG might be able to detect Fapys originated from some alkylated bases (Muruzabal et al. 2020b). Thus, as expected, hAAG-sensitive sites were only found in cells treated with alkylating agents (i.e., MMS and EMS) (Fig. 1b, c, respectively) although with different levels of sensitivity. Indeed, MMS-induced lesions were detected by hAAG enzyme with high sensitivity from the lowest MMS concentration onwards (Fig. 1b), whereas EMS-induced lesions were only revealed at the highest EMS concentration tested (Fig. 1c). This may be explained because although both agents alkylate purine bases, MMS induces 3-methyladenines and 7-methylguanines and EMS induces 3-ethyladenines and 7-ethylguanines (Beranek 1990). As expected, no response was observed with the other compounds. We previously analyzed the specificity of hAAG using the Glyco-SPOT assay, demonstrating its activity for ethenoadenines and hypoxanthine, but we could not test the enzyme with alkylated DNA lesions since they are not included in the assay (Muruzabal et al. 2020b).

Overall, no enzyme detected lesions induced by either cross-linking agents (CisPt and Mit. C) or bulky adducts induced by BPDE. Regarding cross-links, we modified the comet assay for its detection by inducing similar levels of breakage (SBs) of DNA in all samples (after the respective compound treatments) by exposure to H_2_O_2_ to establish a known level of DNA damage (i.e., approximately 40–50% of DNA in tail). Thus, when cross-links are present in DNA, a retardation in DNA migration is caused and comet tails will appear shorter compared to control samples, that will show the expected amount of DNA damage. In this study, we were able to specifically detect the effects of the two cross-linking agents employed, CisPt and Mit. C (Fig. 2d and f, respectively). CisPt induces mainly intra-strand cross-links, but also ICLs and DNA–protein cross-links (Zamble and Lippard 1995; Sanderson et al. 1996); and Mit. C induces ICLs (Tomasz 1994). It should be noted that the significant decrease in the number of SBs found after treatments with 6.66 and 13.33 µM of CisPt and 0.3 µM of Mit. C were obtained at cytotoxic concentrations (RSG of 39, 12, and 50%, respectively). A non-significant decrease was already detectable at non-cytotoxic concentrations with both compounds. As expected, no effect was observed with the genotoxic compounds with other mechanisms of action or with the non-genotoxic compounds, although we found a slight and non-significant concentration-dependent increase in SBs levels of cells treated with Triton X-100, especially at the highest concentration tested (0.1 mM) which is probably due to the high toxicity levels (RSG of 7%) (Fig. 2g).

As aforementioned, we did not detect DNA adducts induced by BPDE with any of the modifications employed in this study. BPDE is the ultimate and DNA reactive metabolite of benzo[a]pyrene (B(a)P) and responsible of B(a)P carcinogenesis (reviewed in Shimada 2006). Interestingly, Azqueta et al. (2013a), reported that the Fpg-modified comet assay increased the sensitivity of the assay towards B(a)P-derived lesions in TK-6 after bioactivation with S9 fraction. This can be explained as it has been shown that during B(a)P metabolism reactive oxygen species may be formed (Flowers et al. 1997), which are detectable by Fpg. When the metabolite BPDE alone is used, it is expected to selectively induce bulky adducts in DNA without inducing radical oxygen species, and thus no DNA damage is expected to be detected with the Fpg-modified comet assay. The bacterial enzyme uvrABC, responsible for nucleotide excision repair system (NER) in prokaryotes is active towards a wide range of substrates including bulky DNA adducts (Sancar and Sancar 1988), and it was employed in combination with the comet assay (Dušinská and Collins 1996), but has not given satisfactory results. However, it is possible to detect these lesions by combining the comet assay with the use of DNA-repair inhibitors, such as aphidicolin, hydroxyurea, and 1-β-D-arabinofuranosylcytosine (Gedik et al. 1992; Martin et al. 1999; Jansen et al. 2001; Speit et al. 2004; Güerci et al. 2009; Vande Loock et al. 2010); although some authors recently hypothesize that this method may also trap BER intermediates, thereby reducing the sensitivity of the assay to detect bulky adducts (Ngo et al. 2020).

The use of the same enzyme-reaction buffer along with the medium-throughput format of 12 minigels/slide of the comet assay facilitated the screening of different lesions in a single assay, as each experiment was much less time-consuming. Although both modifications employed (enzymes and cross-links detection) were performed sequentially, it is completely reasonable to integrate the modifications on a single experiment by including an extra slide in each experiment for the second treatment with H_2_O_2_ for cross-links detection. Indeed, we have already prepared and successfully applied a protocol including both modifications within a single-integrated experiment.

Overall, we specifically detected oxidized and alkylated bases, and cross-links by including different DNA glycosylases in the comet assay as well as the comet assay modified for cross-links detection, respectively. The genotoxic mechanisms of action are of great biological significance, as for instance some oxidized and alkylated bases are potentially mutagenic (Grollman and Moriya 1993; Shrivastav et al. 2009) and cross-linking agents are typically clastogenic (reviewed in Noll et al. 2006). Indeed, nowadays some of these mechanisms of action already have an impact in regulatory decision-making. Directly DNA-damaging compounds are considered non-threshold carcinogens, and thus risk exists at any level of exposure, whereas indirect DNA damage through oxidative stress has threshold effect related with dose, and thus health-based guidance values can be established (EFSA 2005).

Regarding AOPs, in the AOP-Wiki, which is supported by the OECD, some of these DNA lesions are included within its framework as MIEs or KEs (e.g., DNA alkylation or oxidation) which are linked to AO such as heritable mutations in offspring or chromosomal aberrations (AOPWiki 2020). As the number of AOPs is constantly increasing, it is of great importance to develop reliable tools and methods that allow the detection and measurement of the KEs. In this context, the comet assay modifications evaluated in this study are promising tools for in vitro genotoxicity assessment focused on the detection of different mechanisms of action, as we were able to detect and differentiate with high specificity oxidizing, alkylating and cross-linking agents.

## Data Availability

All data generated or analyzed during this study are included in this published article.
